# Disseminated Kaposi Sarcoma With Gastrointestinal Involvement and Primary Effusion Lymphoma in an Untreated HIV Patient: A Case Report

**DOI:** 10.7759/cureus.100770

**Published:** 2026-01-04

**Authors:** Amro Al Radaideh, Amritpal Jagra, Murad Qirem, Ruhma Ali, Ahmad Habbas, Shahd Yaghi, Samer Jumean, Ismail Althunibat, Richard Miller, Hari O Sharma, Nirav Mistry

**Affiliations:** 1 Internal Medicine, Saint Michael's Medical Center, Newark, USA; 2 Pulmonology, Saint Michael's Medical Center, Newark, USA; 3 ICU/Critical Care, Hackensack Meridian Southern Ocean Medical Center, Manahawkin, USA; 4 Intensive Care Unit, Saint Michael's Medical Center, Newark, USA; 5 Critical Care, Saint Michael's Medical Center, Newark, USA

**Keywords:** aids, gastrointestinal involvement, hhv-8, hiv, kaposi sarcoma, pleural effusion, primary effusion lymphoma

## Abstract

Kaposi sarcoma (KS) and primary effusion lymphoma (PEL) are human herpesvirus-8 (HHV-8)-associated malignancies that occur predominantly in individuals with advanced, untreated HIV. We report a 30-year-old male patient with untreated HIV who presented with progressive dyspnea, cough, abdominal pain, and widespread violaceous skin lesions. Imaging revealed bilateral pleural effusions and extensive lymphadenopathy. Biopsies confirmed disseminated KS involving the skin, lymph nodes, and gastrointestinal tract, while pleural fluid cytology and immunophenotypic analysis were diagnostic of PEL. Despite initiation of antiretroviral therapy and chemotherapy, the patient developed refractory effusions, pancytopenia, and ultimately multiorgan and respiratory failure. This case highlights the aggressive nature of dual HHV-8-driven malignancies in untreated HIV patients and underscores the importance of early diagnosis and prompt antiretroviral initiation.

## Introduction

Kaposi sarcoma (KS) is a vascular neoplasm caused by human herpesvirus‑8 (HHV‑8) and is strongly associated with advanced HIV infection [1]. It remains one of the most common AIDS‑defining malignancies [2]. Primary effusion lymphoma (PEL), another HHV‑8-associated tumor [3-6], classically presents as malignant effusions without a discrete mass and typically arises in the setting of profound immunosuppression.

The coexistence of KS and PEL is rare [5] and reflects extensive HHV‑8-mediated oncogenesis. Both malignancies share viral mechanisms that promote angiogenesis, immune evasion, and cellular proliferation. Early recognition and aggressive management, including antiretroviral therapy (ART) and chemotherapy, are essential, although outcomes remain poor when the disease is already disseminated.

We present a case of concurrent disseminated KS with gastrointestinal involvement and PEL in an untreated HIV‑positive man.

## Case presentation

A 30-year-old man with HIV diagnosed three years earlier, who had declined ART due to a combination of denial of his diagnosis and lack of understanding of the disease and its treatment, presented with worsening dyspnea, a persistent nonproductive cough, intermittent upper abdominal pain, nausea, and multiple non-bloody episodes of vomiting. Over the preceding two months, he noted progressive dark purplish lesions over his face, trunk, and extremities.

He appeared thin and chronically ill but alert. His body mass index (BMI) was 19 kg/m². On presentation, vital signs revealed a blood pressure of 98/55 mmHg, heart rate of 110 beats per minute, temperature of 37.5°C, respiratory rate of 15 breaths per minute, and oxygen saturation within normal limits on room air. Physical examination revealed multiple violaceous papules and nodules across the face, trunk, and extremities, along with bilateral inguinal and axillary lymphadenopathy. Breath sounds were diminished bilaterally.

Laboratory findings

Pancytopenia was present, including hemoglobin 7.4 g/dL (reference range: 13.5-17.5 g/dL), WBC 2.1 ×10³/µL (reference range: 4.0-11.0 ×10³/µL), and platelets 84 ×10³/µL (reference range: 150-450 ×10³/µL). CD4 count was 77 cells/µL (reference range: 500-1500 cells/µL; CD4:CD8 ratio reference range: ~1.0-4.0; patient ratio: 0.03). HIV-1 viral load was markedly elevated at 166,974 copies/mL. LDH was elevated at 480 U/L (reference range: 140-280 U/L), and mild transaminitis was noted.

Imaging studies

**Figure 1:**
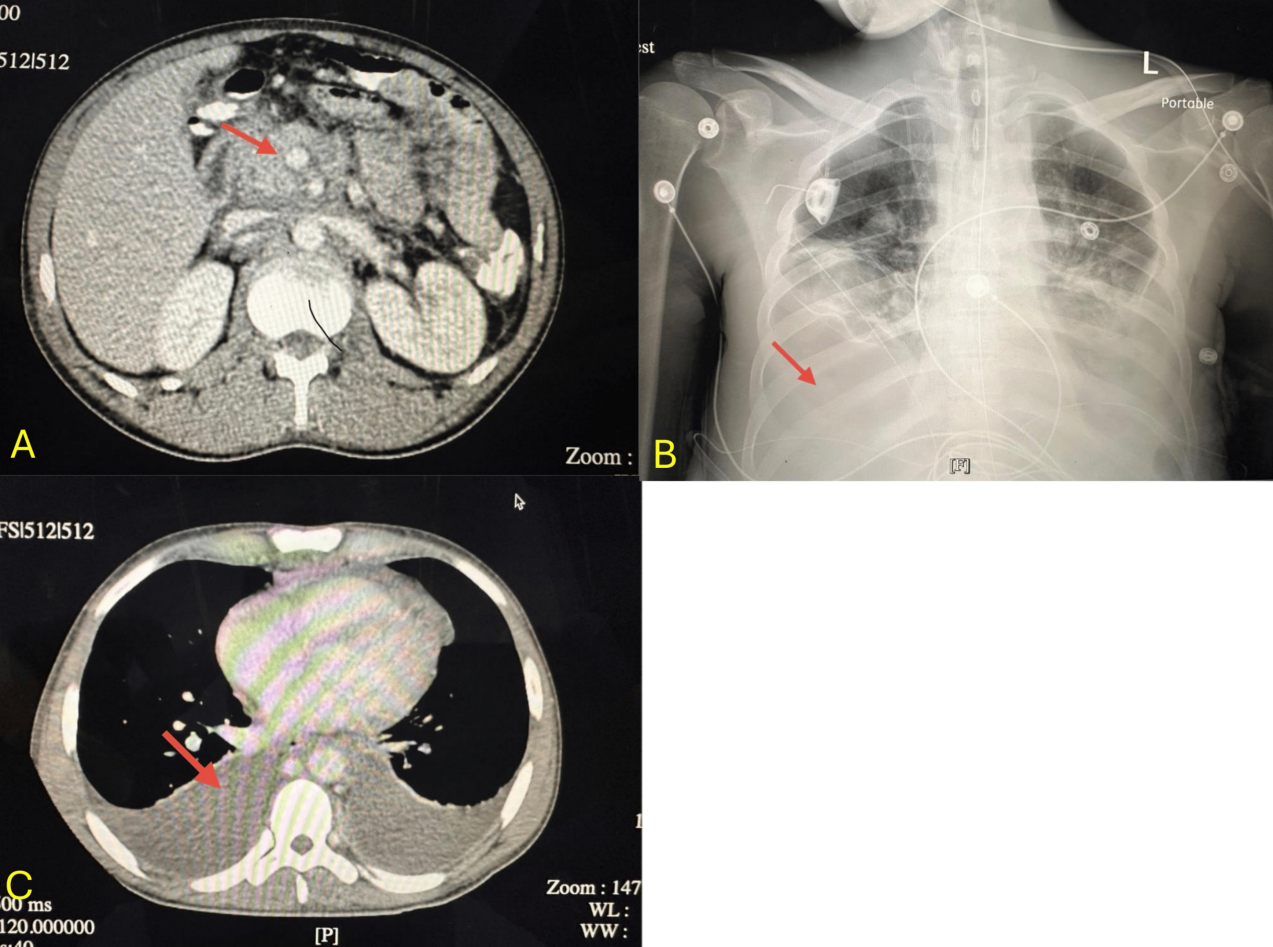
A: CT abdomen/pelvis showing lymphadenopathy. B: Chest X-ray showing bilateral pleural effusions. C: CT chest confirming pleural effusions.

CT abdomen/pelvis (Figure 1A) demonstrated extensive retroperitoneal and inguinal lymphadenopathy with mild hepatomegaly. Chest X‑ray (Figure 1B) revealed moderate bilateral pleural effusions. CT chest (Figure 1C) confirmed large effusions with compressive atelectasis.

Procedures and pathology

**Figure 2:**
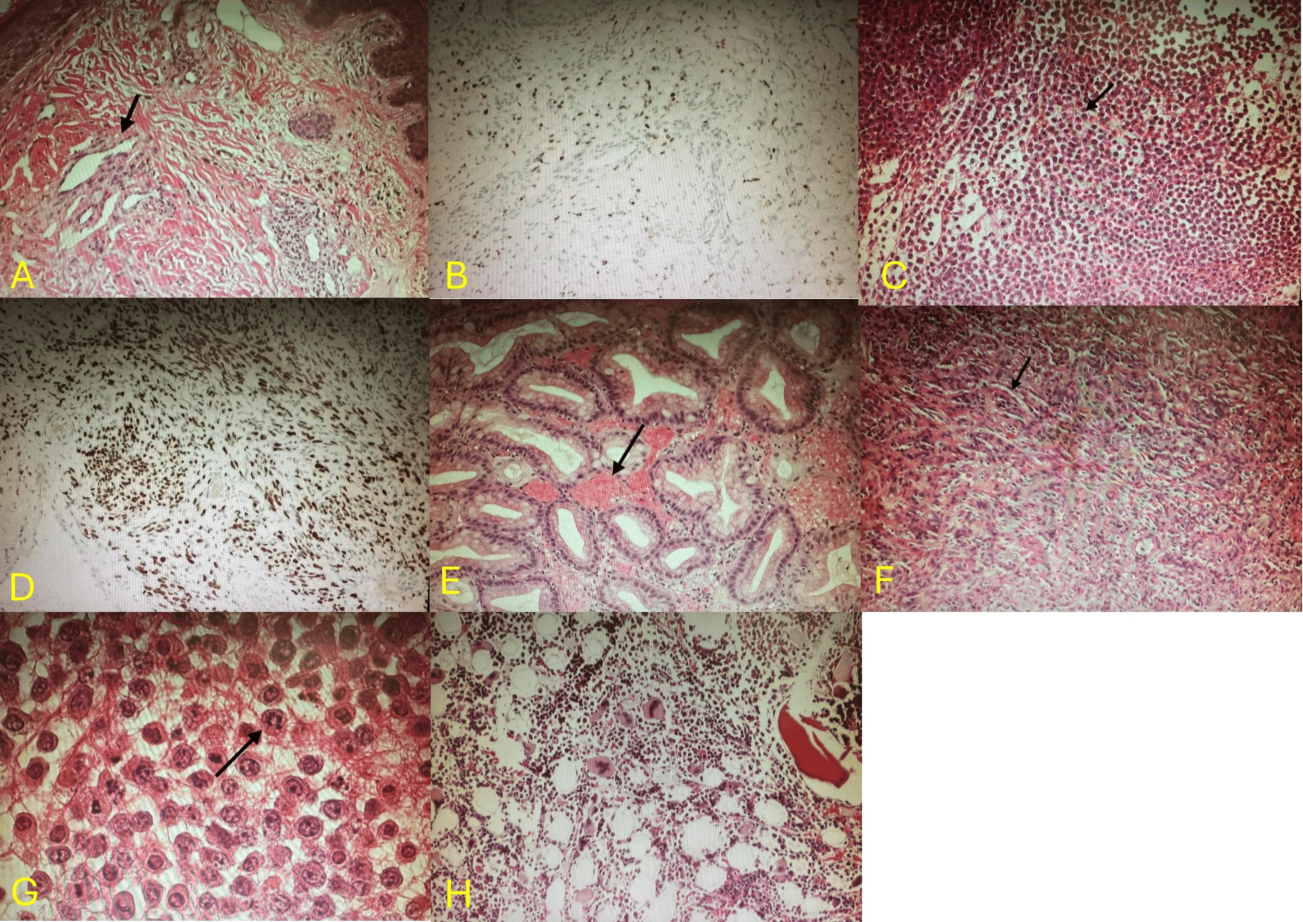
A: Skin biopsy showing abnormal vascular spaces lined with malignant spindle cells (black arrow) consistent with Kaposi sarcoma. B: Immunohistochemical staining of the skin biopsy demonstrating HHV‑8 positivity. C: Lymph node biopsy showing spindle‑cell neoplasm with slit‑like vascular spaces. D: Immunohistochemical staining of the lymph node biopsy showing HHV‑8 positivity. E: Gastric mucosa biopsy showing HHV‑8–positive malignant cells in the lamina propria. F: Low-power view of pleural effusion cytology showing sheets of discohesive atypical lymphocytes with pleomorphism, vesicular chromatin, and prominent nucleoli (black arrow). G: A high-power pleural effusion cytology highlighting discohesive pleomorphic lymphocytes with vesicular chromatin and prominent nucleoli (black arrow), consistent with primary effusion lymphoma. H: Bone marrow biopsy demonstrating no HHV‑8-associated malignancy. HHV-8: Human herpesvirus-8

Skin biopsy (Figures 2A, 2B) demonstrated HHV-8-positive spindle-cell vascular proliferation consistent with KS [1]. Lymph node biopsy (Figures 2C, 2D) confirmed an HHV-8-positive spindle-cell neoplasm [1]. Upper endoscopy identified violaceous gastric nodules; biopsy (Figure 2E) confirmed gastrointestinal KS involvement due to HHV-8-positive spindle cells [3]. Thoracentesis produced serosanguineous exudate; cytology (Figures 2F, 2G) showed large pleomorphic lymphoid cells with prominent nucleoli and abundant cytoplasm. Immunophenotyping demonstrated expression of CD45, CD30, and CD138 with HHV-8 positivity, findings characteristic of PEL rather than KS-associated reactive effusion. The absence of spindle cells or vascular structures within the pleural fluid further supported the diagnosis of PEL. EBV co-infection was confirmed by Epstein-Barr virus-encoded RNA [4,6]. Bone marrow biopsy (Figure 2H) showed trilineage hematopoiesis without evidence of KS or lymphoma. Together, these cytologic, immunophenotypic, and virologic findings fulfill established diagnostic criteria for PEL, thereby confirming a dual presentation of KS and PEL in this patient.

Clinical course

The patient was started on ART and received liposomal doxorubicin followed by CHOP chemotherapy. Due to recurrent pleural effusions, bilateral PleurX catheters were placed. His course was complicated by refractory pancytopenia, hypotension, and ultimately multiorgan failure with progressive respiratory decline. Comfort‑focused care was initiated, and he passed away shortly thereafter.

## Discussion

This case illustrates advanced AIDS-related KS with cutaneous, pulmonary, lymphatic, and gastrointestinal involvement. Pulmonary KS, although uncommon, can mimic opportunistic infections and requires HHV-8 confirmation in cytology [5,7,8]. Prior studies have shown that pulmonary KS often presents with nonspecific respiratory symptoms and pleural effusions, similar to this patient’s presentation, and may be misdiagnosed as infections or heart failure, delaying treatment [7,8].

Gastrointestinal KS may initially be silent [3] but can cause abdominal pain, nausea, vomiting, or gastrointestinal bleeding. In published reports, gastrointestinal involvement is detected in up to 40% of patients with disseminated KS, though endoscopic lesions may be missed without biopsy [3]. Our patient’s gastric involvement aligns with these findings and highlights the importance of endoscopic evaluation when anemia or abdominal symptoms are present.

PEL is another HHV-8-associated malignancy [4,6], presenting as lymphomatous effusions without mass formation. Reported median survival for PEL remains poor, typically ranging from approximately 4 to 6 months, particularly in patients with advanced HIV infection and CD4 counts below 100 cells/µL, as observed in this case [4,6]. Literature indicates that PEL carries a poor prognosis, with median survival often less than six months even with chemotherapy, particularly in untreated HIV [4,6]. Similar to previously reported cases, this patient had profound immunosuppression and EBV co-infection, both of which are strongly associated with PEL pathogenesis and worse outcomes [4,6].

Importantly, dual presentation of KS and PEL is rare but has been described in isolated case reports and is thought to reflect extensive HHV-8-driven oncogenesis in the context of severe immunosuppression [6]. Similar cases in the literature describe rapid clinical deterioration and limited survival despite initiation of ART and chemotherapy, underscoring the aggressive behavior of concurrent HHV-8-associated malignancies [4,6]. Outcomes in such cases are uniformly poor, with rapid clinical decline despite ART and chemotherapy.

In patients with advanced HIV presenting with pleural effusions and systemic symptoms, the differential diagnosis includes KS-associated effusion, opportunistic infections such as tuberculosis, and other HIV-associated lymphomas. In this case, the presence of large atypical lymphoid cells with HHV-8 positivity, a characteristic immunophenotype (CD45, CD30, CD138), EBV co-infection, and the absence of spindle cells or vascular structures in pleural fluid favored a diagnosis of PEL over KS-related effusion or infectious etiologies. Published therapeutic strategies suggest that earlier ART initiation and improved immune reconstitution may improve outcomes, emphasizing the need for earlier HIV diagnosis and treatment [1,2,9].

Early detection, initiation of ART, and coordinated oncologic care are essential in managing HHV-8-associated malignancies [9], although prognosis remains poor in patients with disseminated dual HHV-8-related tumors.

## Conclusions

This case underscores the severe and rapidly progressive nature of HHV-8-associated malignancies in the setting of untreated HIV infection. The simultaneous presence of disseminated KS and PEL highlights the profound immunosuppression required for dual tumor emergence and the shared viral mechanisms driving their pathogenesis. Clinicians should maintain a high index of suspicion for multiple HHV-8-related malignancies when encountering unexplained effusions, mucocutaneous lesions, or gastrointestinal abnormalities in patients with advanced HIV. Early initiation of ART, timely diagnostic evaluation, and coordinated multidisciplinary management remain critical to improving outcomes, although prognosis is often poor once widespread disease has developed.
